# 1-Methyl-3-(4-chloro­benzo­yl)imidazo[1,2-*a*]pyridin-1-ium-2-olate

**DOI:** 10.1107/S1600536811039614

**Published:** 2011-09-30

**Authors:** Victor B. Rybakov, Eugene V. Babaev

**Affiliations:** a Departament of Chemistry, Moscow State University, 119992 Moscow, Russian Federation

## Abstract

In the mol­ecule of the title compound, C_15_H_11_ClN_2_O_2_, the nine-membered heterobicycle is approximately planar [largest deviation from least-squares plane = 0.012 (2) Å] and forms a dihedral angle of 51.14 (8)° with the plane of the 4-chloro­phenyl group. There is a non-classical intra­molecular hydrogen bond between the pyridine α-H atom and the O atom of the benzoyl group. The crystal structure is stabilized by weak C—H⋯O and C—H⋯Cl inter­actions involving the ‘olate’ O atom and the Cl atom attached to the benzoyl group as acceptors.

## Related literature

For related structures, see: Friedman *et al.* (1978 ▶); Rybakov *et al.* (1999 ▶, 2000*a* ▶,*b* ▶, 2001 ▶, 2002 ▶). For the synthesis of 1-methyl-2-oxo-2,3-dihydro­imidazopyridinium perchlorate, see: Sych & Gorb (1976 ▶). For a description of the Cambridge Structural Database, see: Allen (2002 ▶).
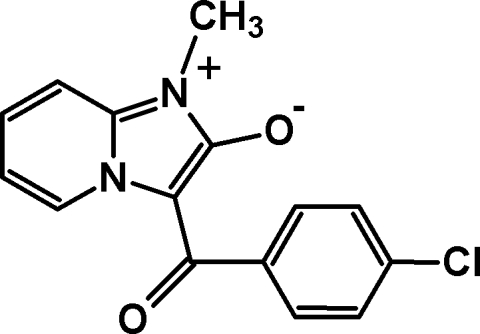


## Experimental

### 

#### Crystal data

C_15_H_11_ClN_2_O_2_
*M* *_r_* = 286.71
Monoclinic, 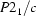

*a* = 8.190 (8) Å
*b* = 13.914 (3) Å
*c* = 11.675 (4) Å
β = 102.38 (2)°
*V* = 1299.5 (14) Å^3^
*Z* = 4
Mo *K*α radiation
μ = 0.30 mm^−1^
*T* = 295 K
0.30 × 0.30 × 0.30 mm

#### Data collection

Enraf–Nonius CAD-4 diffractometer
2675 measured reflections
2546 independent reflections
1486 reflections with *I* > 2σ(*I*)
*R*_int_ = 0.042
1 standard reflections every 200 reflections  intensity decay: 2%

#### Refinement

*R*[*F* ^2^ > 2σ(*F* ^2^)] = 0.044
*wR*(*F* ^2^) = 0.107
*S* = 0.94
2546 reflections
182 parameters
H-atom parameters constrained
Δρ_max_ = 0.15 e Å^−3^
Δρ_min_ = −0.26 e Å^−3^

### 

Data collection: *CAD-4 Software* (Enraf–Nonius, 1994) ▶; cell refinement: *CAD-4 Software* ▶; data reduction: *XCAD4* (Harms & Wocadlo, 1995 ▶); program(s) used to solve structure: *SHELXS97* (Sheldrick, 2008 ▶); program(s) used to refine structure: *SHELXL97* (Sheldrick, 2008 ▶); molecular graphics: *ORTEP-3* (Farrugia, 1997 ▶); software used to prepare material for publication: *WinGX* (Farrugia, 1999 ▶).

## Supplementary Material

Crystal structure: contains datablock(s) global, I. DOI: 10.1107/S1600536811039614/yk2022sup1.cif

Structure factors: contains datablock(s) I. DOI: 10.1107/S1600536811039614/yk2022Isup2.hkl

Supplementary material file. DOI: 10.1107/S1600536811039614/yk2022Isup3.cml

Additional supplementary materials:  crystallographic information; 3D view; checkCIF report

## Figures and Tables

**Table 1:** Hydrogen-bond geometry (Å, °)

*D*—H⋯*A*	*D*—H	H⋯*A*	*D*⋯*A*	*D*—H⋯*A*
C5—H5⋯O30	0.93	2.30	2.863 (3)	119
C8—H8⋯O2^i^	0.93	2.47	3.291 (4)	148
C32—H32⋯Cl34^ii^	0.93	2.93	3.794 (3)	155

Symmetry codes: (i) 
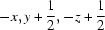
; (ii) 
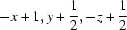
.

**Fig. 1.**
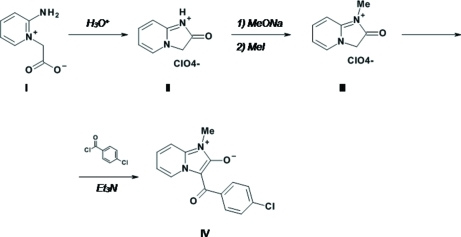
Synthesis path for IV.

**Fig. 2.**
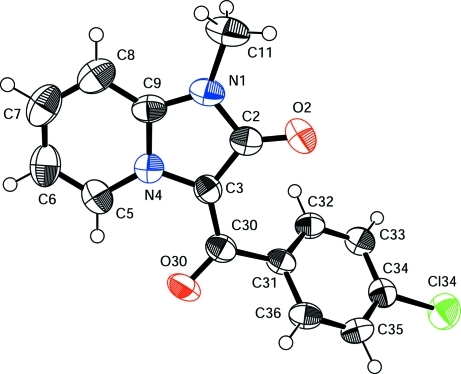
ORTEP-3 (Farrugia, 1997) plot of the molecule IV with the atom numbering scheme. Displacement ellipsoids are shown at the 50% probability level. H atoms are presented as small spheres of arbitrary radius.

## supplementary crystallographic information

### Comment

Early we described crystal structures of "pyridylglycine" I (Rybakov *et al.*, 1999) (Fig. 1) and the product of its cyclocondensation – 2-oxoimidazo[1,2-*a*]pyridine II (Rybakov *et al.*, 2000*a*) (Fig. 1). According to Sych & Gorb (1976), we have also performed selective *N*–methylation of II and investigated the molecular and crystal structures of the resulting salt III (Rybakov *et al.*, 2000*b*) (Fig. 1). In the present paper we continue the sequence I-II-III and report the molecular structure of the acylated derivative of the compound III - the mesoionic 1-methyl-3-(*p*–chlorobenzoyl)imidazo[1,2-*a*] pyridinium-2-olate IV (Fig. 1). The acylation of III was performed by using of 4-chlorobenzoyl chloride in the presence of triethylamine leading to green crystals of the derivative IV with the 60% yield.

The molecular structure of the mesoionic compound IV (Fig. 2) displays some remarkable features early observed for analogous fused imidazopyridines (Friedman *et al.*, 1978) and oxazolopyridines (Rybakov *et al.*, 2001; Rybakov *et al.*, 2002). In particular, in the moiety O10═ C30—C3—C2═O2 the bonds length C3—C30 and C2—C3 correspond to single bonds (~1.43 Å), whereas the bonds length C30═O30 and C2═ O2 (~1.23 Å) correspond to double bonds, thus displaying the unusual ylide-like pattern of the imidazolone fragment. On the other hand, the sequence C5═C6–C7═C8 displays alternation of the bonds length, thus corresponding to quasi-diene fragment of the pyridine ring. These facts seem to be common to the entire class of azolopyridinium-2-olates. The intramolecular interaction C5—H5···O30 with parameters H5···O30 = 2.296 Å, C5···O30 = 2.863 (3)Å and angle C5—H5···O30 = 118.83° (Table 1) is found. The molecules in crystal are linked by weak intermolecular interactions: C8—H8···O2^i^ with parameters H8···O2^i^ = 2.466 Å, C8···O2^i^ = 3.291 (4)Å and angle C8—H8···O2^i^ = 147.86°; C32—H32···Cl34^ii^ with parameters H32···Cl34^ii^ = 2.931 Å, C32···Cl34 = 3.794 (3)Å and angle C32—H32···Cl34^ii^ = 154.93°. Symmetry codes: (i) -*x*, *y* + 1/2, -*z* + 1/2; (ii) -*x* + 1, *y* + 1/2, -*z* + 1/2.

### Experimental

1-Methyl-2-oxo-2,3-dihydroimidazopyridinium perchlorate III was obtained as described by Sych & Gorb (1976) (Fig. 2). In order to obtain 1-methyl-3-(4-chlorobenzoyl)imidazo[1,2-*a*]pyridinium-2-olate IV, triethylamine (2.24 ml, 0.016 mol) was added slowly to the solution of 2.0 g (8 mmol) III in 10 ml of acetonitrile, and 1.4 g (8 mmol) of 4-chlorobenzoil chloride was added to the obtained mixture. The reaction flask was stirred at room temperature for 1 h and then kept overnight. The precipitate was filtered and recrystallized from isopropyl alcohol. The yield was 1.3 g (60%). *M*.p. 479–481 K. ^1^H NMR spectra (*DMSO*-d_6_, p.p.m.): 9.96 (d, 1H, 9-H), 7.85 (dd, 1H, 8-H), 7.68 (m, 2H, *p*-Cl*Ph*), 7.61 (d, 1H, 6-H), 7.44 (m, 2H, *p*-Cl*Ph*), 7.34 (dd, 1H, 7-H), 3.38 (s, 3H, 4-*Me*). The numbering of protons is given according to the atoms numbering on Fig. 1.

### Refinement

All the hydrogen atoms in the molecule were placed geometrically and allowed to ride on their parent atoms with C—H distance in the range 0.93Å and 0.96Å and with *U*_iso_(H) = 1.5*U*_eq_(C) for CH_3_ group and *U*_iso_(H) = 1.2*U*_eq_(C) for the aryl groups.

### Crystal data

**Table:**

C_15_H_11_ClN_2_O_2_	*F*(000) = 592
*M**_r_* = 286.71	*D*_x_ = 1.465 Mg m^−^^3^
Monoclinic, *P*2_1_/*c*	Melting point = 479–481 K
Hall symbol: -P 2ybc	Mo *K*α radiation, λ = 0.71073 Å
*a* = 8.190 (8) Å	Cell parameters from 25 reflections
*b* = 13.914 (3) Å	θ = 13.0–14.8°
*c* = 11.675 (4) Å	µ = 0.30 mm^−^^1^
β = 102.38 (2)°	*T* = 295 K
*V* = 1299.5 (14) Å^3^	Prism, green
*Z* = 4	0.30 × 0.30 × 0.30 mm

### Data collection

**Table:**

Enraf–Nonius CAD-4 diffractometer	*R*_int_ = 0.042
Radiation source: fine-focus sealed tube	θ_max_ = 26.0°, θ_min_ = 2.3°
graphite	*h* = −10→9
non–profiled ω scans	*k* = 0→17
2675 measured reflections	*l* = 0→14
2546 independent reflections	1 standard reflections every 200 reflections
1486 reflections with *I* > 2σ(*I*)	intensity decay: 2%

### Refinement

**Table:**

Refinement on *F*^2^	Primary atom site location: structure-invariant direct methods
Least-squares matrix: full	Secondary atom site location: difference Fourier map
*R*[*F*^2^ > 2σ(*F*^2^)] = 0.044	Hydrogen site location: inferred from neighbouring sites
*wR*(*F*^2^) = 0.107	H-atom parameters constrained
*S* = 0.94	*w* = 1/[σ^2^(*F*_o_^2^) + (0.0433*P*)^2^] where *P* = (*F*_o_^2^ + 2*F*_c_^2^)/3
2546 reflections	(Δ/σ)_max_ < 0.001
182 parameters	Δρ_max_ = 0.15 e Å^−^^3^
0 restraints	Δρ_min_ = −0.26 e Å^−^^3^

### Special details

**Table:**

Geometry. All s.u.'s (except the s.u. in the dihedral angle between two l.s. planes) are estimated using the full covariance matrix. The cell s.u.'s are taken into account individually in the estimation of s.u.'s in distances, angles and torsion angles; correlations between s.u.'s in cell parameters are only used when they are defined by crystal symmetry. An approximate (isotropic) treatment of cell s.u.'s is used for estimating s.u.'s involving l.s. planes.
Refinement. Refinement of F^2^ against ALL reflections. The weighted R-factor wR and goodness of fit S are based on F^2^, conventional R-factors R are based on F, with F set to zero for negative F^2^. The threshold expression of F^2^ > 2σ(F^2^) is used only for calculating R-factors(gt) etc. and is not relevant to the choice of reflections for refinement. R-factors based on F^2^ are statistically about twice as large as those based on F, and R-factors based on ALL data will be even larger.

### Fractional atomic coordinates and isotropic or equivalent isotropic displacement parameters (Å^2^)

**Table:**

	*x*	*y*	*z*	*U*_iso_*/*U*_eq_	
N1	0.0863 (3)	0.54749 (15)	0.22101 (19)	0.0505 (6)	
C2	0.1226 (3)	0.45544 (18)	0.1820 (2)	0.0449 (6)	
O2	0.0815 (2)	0.38150 (13)	0.22548 (16)	0.0594 (5)	
C3	0.2076 (3)	0.47398 (16)	0.0895 (2)	0.0424 (6)	
N4	0.2102 (2)	0.57470 (13)	0.07555 (17)	0.0416 (5)	
C5	0.2755 (3)	0.62878 (19)	−0.0013 (2)	0.0521 (7)	
H5	0.3244	0.5997	−0.0574	0.063*	
C6	0.2675 (4)	0.7264 (2)	0.0060 (3)	0.0646 (9)	
H6	0.3118	0.7641	−0.0457	0.078*	
C7	0.1947 (4)	0.7704 (2)	0.0887 (3)	0.0690 (9)	
H7	0.1903	0.8371	0.0920	0.083*	
C8	0.1292 (4)	0.71635 (19)	0.1653 (3)	0.0609 (8)	
H8	0.0801	0.7453	0.2213	0.073*	
C9	0.1377 (3)	0.61773 (18)	0.1577 (2)	0.0469 (6)	
C11	−0.0063 (4)	0.5623 (2)	0.3124 (3)	0.0725 (9)	
H11A	0.0696	0.5797	0.3840	0.109*	
H11B	−0.0636	0.5041	0.3240	0.109*	
H11C	−0.0863	0.6129	0.2895	0.109*	
C30	0.2748 (3)	0.40950 (17)	0.0162 (2)	0.0459 (6)	
O30	0.3115 (3)	0.43543 (13)	−0.07607 (15)	0.0659 (6)	
C31	0.3064 (3)	0.30759 (17)	0.0552 (2)	0.0390 (6)	
C32	0.3762 (3)	0.28280 (17)	0.1699 (2)	0.0451 (6)	
H32	0.3955	0.3304	0.2271	0.054*	
C33	0.4177 (3)	0.18918 (18)	0.2012 (2)	0.0463 (6)	
H33	0.4654	0.1733	0.2785	0.056*	
C34	0.3869 (3)	0.11962 (17)	0.1154 (2)	0.0450 (6)	
Cl34	0.43791 (11)	0.00078 (5)	0.15427 (8)	0.0725 (3)	
C35	0.3144 (3)	0.14142 (17)	0.0010 (2)	0.0478 (7)	
H35	0.2914	0.0931	−0.0552	0.057*	
C36	0.2766 (3)	0.23515 (18)	−0.0291 (2)	0.0460 (7)	
H36	0.2305	0.2507	−0.1068	0.055*	

### Atomic displacement parameters (Å^2^)

**Table:**

	*U*^11^	*U*^22^	*U*^33^	*U*^12^	*U*^13^	*U*^23^
N1	0.0450 (14)	0.0641 (14)	0.0448 (14)	0.0060 (11)	0.0150 (12)	−0.0039 (12)
C2	0.0378 (16)	0.0555 (15)	0.0389 (15)	0.0004 (12)	0.0023 (13)	−0.0033 (13)
O2	0.0603 (13)	0.0647 (12)	0.0568 (13)	−0.0035 (10)	0.0208 (11)	0.0127 (10)
C3	0.0415 (15)	0.0452 (13)	0.0396 (15)	0.0000 (11)	0.0071 (13)	0.0030 (11)
N4	0.0339 (12)	0.0468 (11)	0.0409 (12)	0.0017 (9)	0.0012 (10)	0.0011 (10)
C5	0.0418 (17)	0.0627 (17)	0.0506 (17)	0.0017 (13)	0.0071 (14)	0.0112 (14)
C6	0.050 (2)	0.0561 (17)	0.082 (2)	−0.0012 (14)	0.0015 (18)	0.0165 (16)
C7	0.056 (2)	0.0515 (17)	0.089 (3)	0.0079 (15)	−0.0079 (19)	−0.0010 (18)
C8	0.0476 (19)	0.0582 (17)	0.072 (2)	0.0084 (14)	0.0022 (17)	−0.0111 (16)
C9	0.0372 (16)	0.0568 (15)	0.0460 (17)	0.0101 (12)	0.0071 (13)	−0.0063 (13)
C11	0.069 (2)	0.098 (2)	0.057 (2)	0.0132 (18)	0.0286 (18)	−0.0073 (18)
C30	0.0430 (16)	0.0554 (16)	0.0382 (16)	−0.0002 (12)	0.0060 (13)	0.0004 (12)
O30	0.0985 (17)	0.0675 (12)	0.0383 (11)	0.0132 (11)	0.0289 (12)	0.0086 (10)
C31	0.0336 (14)	0.0519 (14)	0.0291 (14)	0.0011 (11)	0.0012 (11)	−0.0001 (11)
C32	0.0471 (17)	0.0500 (14)	0.0359 (15)	−0.0021 (12)	0.0037 (13)	−0.0056 (12)
C33	0.0409 (16)	0.0557 (15)	0.0392 (15)	0.0044 (12)	0.0019 (12)	0.0035 (13)
C34	0.0362 (15)	0.0475 (14)	0.0536 (17)	0.0070 (11)	0.0149 (14)	0.0050 (13)
Cl34	0.0791 (6)	0.0543 (4)	0.0870 (7)	0.0176 (4)	0.0246 (5)	0.0101 (4)
C35	0.0469 (17)	0.0510 (15)	0.0434 (16)	0.0013 (12)	0.0055 (13)	−0.0132 (13)
C36	0.0412 (16)	0.0596 (16)	0.0343 (15)	0.0038 (12)	0.0016 (13)	−0.0043 (12)

### Geometric parameters (Å, °)

**Table:**

Cl34—C34	1.742 (2)	C5—H5	0.9300
N4—C5	1.365 (3)	C30—O30	1.233 (3)
N4—C9	1.370 (3)	C30—C31	1.495 (3)
N4—C3	1.412 (3)	C31—C32	1.382 (3)
C2—C3	1.428 (3)	C31—C36	1.393 (3)
C3—C30	1.429 (3)	C32—C33	1.376 (3)
C2—O2	1.226 (3)	C32—H32	0.9300
C2—N1	1.412 (3)	C33—C34	1.377 (3)
N1—C9	1.346 (3)	C33—H33	0.9300
N1—C11	1.450 (3)	C34—C35	1.374 (3)
C9—C8	1.378 (3)	C35—C36	1.369 (3)
C8—C7	1.364 (4)	C35—H35	0.9300
C8—H8	0.9300	C36—H36	0.9300
C7—C6	1.382 (4)	C11—H11A	0.9600
C7—H7	0.9300	C11—H11B	0.9600
C6—C5	1.364 (4)	C11—H11C	0.9600
C6—H6	0.9300		
			
C5—N4—C9	120.6 (2)	O30—C30—C3	122.5 (2)
C5—N4—C3	129.8 (2)	O30—C30—C31	119.0 (2)
C9—N4—C3	109.5 (2)	C3—C30—C31	118.5 (2)
N4—C3—C2	106.7 (2)	C32—C31—C36	118.5 (2)
N4—C3—C30	122.5 (2)	C32—C31—C30	122.7 (2)
C2—C3—C30	130.7 (2)	C36—C31—C30	118.6 (2)
O2—C2—N1	122.1 (2)	C33—C32—C31	121.4 (2)
O2—C2—C3	133.4 (2)	C33—C32—H32	119.3
N1—C2—C3	104.5 (2)	C31—C32—H32	119.3
C9—N1—C2	111.7 (2)	C32—C33—C34	118.4 (2)
C9—N1—C11	125.1 (2)	C32—C33—H33	120.8
C2—N1—C11	123.1 (2)	C34—C33—H33	120.8
N1—C9—N4	107.5 (2)	C35—C34—C33	121.7 (2)
N1—C9—C8	131.5 (3)	C35—C34—Cl34	119.5 (2)
N4—C9—C8	121.0 (3)	C33—C34—Cl34	118.8 (2)
C7—C8—C9	118.4 (3)	C36—C35—C34	119.1 (2)
C7—C8—H8	120.8	C36—C35—H35	120.4
C9—C8—H8	120.8	C34—C35—H35	120.4
C8—C7—C6	120.2 (3)	C35—C36—C31	120.8 (2)
C8—C7—H7	119.9	C35—C36—H36	119.6
C6—C7—H7	119.9	C31—C36—H36	119.6
C5—C6—C7	121.3 (3)	N1—C11—H11A	109.5
C5—C6—H6	119.4	N1—C11—H11B	109.5
C7—C6—H6	119.4	N1—C11—H11C	109.5
C6—C5—N4	118.5 (3)	H11A—C11—H11B	109.5
C6—C5—H5	120.7	H11A—C11—H11C	109.5
N4—C5—H5	120.7	H11B—C11—H11C	109.5
			
C5—N4—C3—C2	−179.6 (2)	C8—C7—C6—C5	0.1 (5)
C9—N4—C3—C2	2.5 (3)	C7—C6—C5—N4	−0.2 (4)
C5—N4—C3—C30	−2.0 (4)	C9—N4—C5—C6	0.3 (4)
C9—N4—C3—C30	−179.9 (2)	C3—N4—C5—C6	−177.5 (2)
N4—C3—C2—O2	176.9 (3)	N4—C3—C30—O30	−13.5 (4)
C30—C3—C2—O2	−0.5 (5)	C2—C3—C30—O30	163.5 (3)
N4—C3—C2—N1	−2.5 (3)	N4—C3—C30—C31	164.6 (2)
C30—C3—C2—N1	−179.8 (3)	C2—C3—C30—C31	−18.4 (4)
O2—C2—N1—C9	−177.7 (2)	O30—C30—C31—C32	136.1 (3)
C3—C2—N1—C9	1.8 (3)	C3—C30—C31—C32	−42.1 (4)
O2—C2—N1—C11	−2.0 (4)	O30—C30—C31—C36	−38.7 (4)
C3—C2—N1—C11	177.5 (2)	C3—C30—C31—C36	143.1 (2)
C2—N1—C9—N4	−0.3 (3)	C36—C31—C32—C33	0.7 (4)
C11—N1—C9—N4	−175.9 (2)	C30—C31—C32—C33	−174.1 (2)
C2—N1—C9—C8	−179.5 (3)	C31—C32—C33—C34	−0.5 (4)
C11—N1—C9—C8	4.9 (5)	C32—C33—C34—C35	−0.9 (4)
C5—N4—C9—N1	−179.5 (2)	C32—C33—C34—Cl34	−179.66 (19)
C3—N4—C9—N1	−1.4 (3)	C33—C34—C35—C36	2.0 (4)
C5—N4—C9—C8	−0.2 (4)	Cl34—C34—C35—C36	−179.18 (19)
C3—N4—C9—C8	177.9 (3)	C34—C35—C36—C31	−1.8 (4)
N1—C9—C8—C7	179.3 (3)	C32—C31—C36—C35	0.5 (4)
N4—C9—C8—C7	0.1 (4)	C30—C31—C36—C35	175.5 (2)
C9—C8—C7—C6	−0.1 (5)		

### Hydrogen-bond geometry (Å, °)

**Table:**

*D*—H···*A*	*D*—H	H···*A*	*D*···*A*	*D*—H···*A*
C5—H5···O30	0.93	2.30	2.863 (3)	119.
C8—H8···O2^i^	0.93	2.47	3.291 (4)	148.
C32—H32···Cl34^ii^	0.93	2.93	3.794 (3)	155.

Symmetry codes:  (i) −*x*, *y*+1/2, −*z*+1/2; (ii) −*x*+1, *y*+1/2, −*z*+1/2.
